# Binding of Rap1 and Riam to Talin1 Fine-Tune β2 Integrin Activity During Leukocyte Trafficking

**DOI:** 10.3389/fimmu.2021.702345

**Published:** 2021-08-19

**Authors:** Thomas Bromberger, Sarah Klapproth, Ina Rohwedder, Jasmin Weber, Robert Pick, Laura Mittmann, Soo Jin Min-Weißenhorn, Christoph A. Reichel, Christoph Scheiermann, Markus Sperandio, Markus Moser

**Affiliations:** ^1^Center for Translational Cancer Research (TranslaTUM), TUM School of Medicine, Technische Universität München, Munich, Germany; ^2^Department of Molecular Medicine, Max Planck Institute of Biochemistry, Martinsried, Germany; ^3^Walter Brendel Center of Experimental Medicine (WBex), Biomedical Center (BMC), Ludwig-Maximilians-Universität München, Martinsried, Germany; ^4^Department of Pathology and Immunology, School of Medicine, University of Geneva, Geneva, Switzerland; ^5^Walter Brendel Centre of Experimental Medicine (WBex), Klinikum der Universität München, Ludwig-Maximilians-Universität München, Munich, Germany; ^6^Department of Otorhinolaryngology, Ludwig-Maximilians-Universität München, Munich, Germany; ^7^Transgenic Core Facility, Max Planck Institute of Biochemistry, Martinsried, Germany

**Keywords:** talin, Riam, Rap1, leukocyte adhesion, leukocyte rolling, integrin activation, leukocyte trafficking

## Abstract

β2 integrins mediate key processes during leukocyte trafficking. Upon leukocyte activation, the structurally bent β2 integrins change their conformation towards an extended, intermediate and eventually high affinity conformation, which mediate slow leukocyte rolling and firm arrest, respectively. Translocation of talin1 to integrin adhesion sites by interactions with the small GTPase Rap1 and the Rap1 effector Riam precede these processes. Using Rap1 binding mutant talin1 and Riam deficient mice we show a strong Riam-dependent T cell homing process to lymph nodes in adoptive transfer experiments and by intravital microscopy. Moreover, neutrophils from compound mutant mice exhibit strongly increased rolling velocities to inflamed cremaster muscle venules compared to single mutants. Using Hoxb8 cell derived neutrophils generated from the mutant mouse strains, we show that both pathways regulate leukocyte rolling and adhesion synergistically by inducing conformational changes of the β2 integrin ectodomain. Importantly, a simultaneous loss of both pathways results in a rolling phenotype similar to talin1 deficient neutrophils suggesting that β2 integrin regulation primarily occurs *via* these two pathways.

## Introduction

Trafficking of neutrophils to sites of inflammation as well as lymphocyte homing to various destinations are fundamental processes of the immune system to defend the host against invaders. In order to leave the blood system, leukocytes follow a defined series of steps, which allow them to transit from a flowing to an adherent state. The leukocyte adhesion cascade includes leukocyte rolling, arrest, crawling and transendothelial migration (1). Integrins, in particular members of the β2 integrin family, contribute to each of these processes upon activation, which may be triggered from surface proteins such as P-selectin glycoprotein ligand-1 (PSGL-1) or chemokine receptors (2–4). These signaling events induce a conformational shift of the integrin´s ectodomain from a low towards an intermediate and eventually high ligand binding affinity, characterized by a bent and extended conformation with closed and open ligand binding pocket, respectively. While intermediate affinity integrin αLβ2 support slow leukocyte rolling, transition to the high affinity conformation is required for firm adhesion (3).

The thermodynamically unfavored extended β2 integrin conformations are stabilized by the binding of talin1 and kindlin-3 to the integrin´s cytoplasmic tail (5). Talin1 is a large cytoplasmic adapter protein, which consists of an N-terminal head domain comprising an atypical FERM domain and a long C-terminal rod domain. While the talin1 rod interacts with a variety of other adapter and signaling molecules as well as the actin cytoskeleton, talin head binding to the cytoplasmic tail of β integrin subunits is crucial for the induction of the active integrin conformations (6, 7). Cytoplasmic talin adopts an auto-inhibited conformation, which is stabilized by intra-molecular interactions between the talin head and rod domains. Therefore, membrane recruitment and release of auto-inhibition are prerequisites for talin1-mediated integrin activation (8–10).

The mechanism of talin recruitment has been in the focus of several recent studies, which led to the identification of different pathways. Interestingly, these pathways differ dependent on the cell type and integrin class. While a series of cell biological studies suggested that a ternary complex consisting of the small membrane-bound GTPase Rap1, its effector Riam and talin is necessary to recruit talin to the membrane (11–13), studies in Riam deficient mice revealed that this complex specifically regulates leukocyte β2 integrins but is not involved in the regulation of other integrin classes expressed in other cells such as platelets (14–16). More recently it has been shown that GTP-bound Rap1 directly binds to the talin head and this interaction is critical for platelet integrin activation (17–20). This pathway seems of general importance as it controls talin activity in the amoeba *Dictyostelium*, flies and mice (17, 21, 22). Mutation of the Rap1 binding site in the talin F0 domain impairs integrin-mediated functions of platelets and neutrophils both *in vitro* and *in vivo* (17, 19). Interestingly, recent studies showed that the F0 binding site synergizes with an additional Rap1 binding site in the structurally similar F1 domain and mutating both Rap1 binding domains causes an additive effect (23–25). It is also important to note that positively charged patches in the talin F2 and F3 domains together with a positively charged loop within the F1 domain interact with membrane lipids and contribute significantly to talin membrane targeting (23, 24, 26). While the Rap1/talin and talin/membrane interactions act synergistically on talin recruitment and integrin activation, mutations of the Riam binding sites in the talin R3 and R8 domains show no cooperative effect on integrin activity and function in fibroblasts (23). So far it has not been analyzed whether and how the more general Rap1/talin and the specific Rap1/Riam/talin pathways cooperate in regulating β2 integrin activity.

In this study, we aimed to decipher the roles of Rap1/talin and Rap1/Riam/talin recruitment pathways in hematopoietic cells by comparing integrin activity and function in Rap1 binding deficient talin knock-in (Tln1^3mut^), Riam deficient (Riam^-/-^) and double mutant (DM; Tln1^3mut^/Riam^-/-^) mice. Our data clearly demonstrate that both pathways synergize to regulate integrin-mediated leukocyte rolling and adhesion by inducing conformational changes of the β2 integrin ectodomain. A simultaneous, genetic block of both pathways results in a phenotype similar to talin1-deficient neutrophils.

## Material and Methods

### Mice

Talin^3mut^ and Riam^-/-^ single mutant mice were described earlier (14, 17). Talin^3mut^/Riam^-/-^ double mutant (DM) mice were generated by mating these single mutant animals. Mice were housed under specific pathogen free conditions. All animal experiments were performed with approval of the District Government of Bavaria (Regierung von Oberbayern, Munich).

### Generation, Culture, and Differentiation of Hoxb8-FL Cells

Hoxb8-FL cells were generated and cultured from bone marrow of WT, Tln1^3mut^, Riam^-/-^ and DM mice as described previously (27, 28).

Hoxb8-FL cells were allowed to differentiate towards neutrophils by keeping them in RPMI1640 supplemented with 10% FBS, penicillin/streptomycin, 2% SCF-containing supernatant, 50 µM β-mercaptoethanol, and 20 ng/ml rmG-CSF (PeproTech) for 4 d.

### CRISPR/Cas9-Mediated Gene Ablation and Expression of Human Integrin β2

CRISPR/Cas9 target sequences were identified with help of the CHOPCHOP (version 3) web tool (29). Guide RNAs to these sequences (Integrin β2: GCGCAAUGUCACGAGGCUGC, Talin1: GGAUCCGCUCACGAAUCAUG) were purchased from Integrated DNA technologies (IDT, Leuven, Belgium). RNA/protein complexes were allowed to form by incubating sgRNAs with TrueCut Cas9 Protein v2 (Thermo Fisher Scientific) for 10 min at room temperature. Subsequently, Hoxb8 cells were electroporated in the presence of the RNP complex using the NEON transfection system (Thermo Fisher Scientific).

To obtain talin1 deficient cells, single cell clones of electroporated cells were generated by limited dilution. Subsequently, knockout clones were identified by PCR and sequencing of the product (forward primer: TTAAATAGGACGGACAGCTTACT, reverse: CTTCACTGTGGCCAAACAGC) and confirmed by Western blotting.

Integrin β2 CRISPR-targeted cells were infected with a pMIGR retroviral vector containing human integrin β2 cDNA by spinoculation. Cells expressing human but not mouse β2 integrin were sorted using a FACSAria™ III sorter (BD Biosciences, Heidelberg, Germany).

### Antibodies

The following antibodies were used for Western blot diluted in 5% milk/TBS-T: mouse anti-Talin (Sigma-Aldrich, Munich, Germany 1:20000), rabbit anti-Riam (Abcam, Berlin, Germany, 1:1000), rabbit anti-Rap1 (Santa Cruz Biotechnology, Heidelberg, Germany, 1:500), rabbit anti-Kindlin-3 (homemade (30); 1:3000), rabbit anti-Lamellipodin (kindly provided by Prof. Gertler, MIT, Cambridge, MA), mouse anti-GAPDH (Merck Millipore, 1:20000), goat anti-mouse-HRP and goat anti-rabbit-HRP (Jackson ImmunoResearch Laboratories, Cambridge, UK 1:15000).

The following antibodies were used at a 1:200 dilution for FACS analysis to determine surface expression levels of relevant integrins and other receptors: hamster anti-mouse CD29-Alexa Fluor 647, rat anti-mouse CD18-APC, hamster anti-mouse CD61-Alexa Fluor 647, rat anti-mouse CD49d-Alexa Fluor 647, rat anti-mouse CD49e-Alexa Fluor 647, rat anti-mouse CD11a-APC, rat anti-mouse CD11b-APC, rat anti-mouse PSGL-1-BV421 (all BD Biosciences), hamster anti-mouse CD49b, rat anti-mouse CD41-PE, rat anti-mouse Gr-1-FITC (Thermo Fisher Scientific), rat anti-human CD18-APC (Biolegend, London, UK).

### Isolation of Primary Neutrophils

Neutrophils were isolated from bone marrow using an EasySep Mouse Neutrophil Enrichment Kit (STEMCELL Technologies, Cologne, Germany) following the manufacturer’s instructions.

### Neutrophil Static Adhesion and Flow Chambers

Neutrophil static adhesion assays were performed with Hoxb8-derived neutrophils as previously described (17) after pre-incubating differentiated cells with 10 mM EDTA for 15 min.

Neutrophil adhesion under flow conditions was assessed in ibidi slides VI 0.1 (Ibidi, Martinsried, Germany) coated with rmP-Selectin (His-tag; R&D systems, Abingdon, UK) and Mouse soluble ICAM-1 (STEMCELL Technologies) with or without rmKC (R&D systems) in coating buffer (20 mM Tris-HCl pH 9.0, 150 mM NaCl, 2 mM MgCl_2_) over night. Neutrophil suspensions of 0.75x10^6^ cells/ml density were perfused through flow chambers at a wall shear stress of 0.3 or 1 dyne/cm^2^ for 10 min using a PHD ULTRA pump (Harvard Apparatus, Holliston, MA, USA). Movies of 10 s were recorded from five different fields of view within the last minute of perfusion using the Evos M7000 life cell imaging system (Thermo Fisher Scientific). ImageJ software was used to analyze velocities of rolling cells as well as the number of adherent cells.

### β2 Integrin Activation Assay

Human β2 integrin expressing Hoxb8 cells were differentiated to neutrophils. Before addition of the reporter antibodies, cells were treated with 10 mM EDTA for 15 min. Stainings with conformation specific antibodies mAb24 and KIM127 were performed in RPMI adhesion medium (RPMI1640 containing 0.1% FBS, penicillin/streptomycin, 50 µM β-mercaptoethanol, 1 µM β-estradiol). Neutrophils were either left untreated or treated with 10 mM EDTA, 0.2 µg/ml TNFα, 10 µM fMLP, 1µg/ml CXCL1 or 1µg/ml PMA and stained with Alexa Fluor 647-labeled mouse anti-human extended conformation specific antibody KIM127 or BV421 conjugated mouse anti-human active conformation specific antibody mAb24 (Biolegend) for 30 min at 37°C. Staining intensities were acquired using a Cytoflex LX flow cytometer (Beckman Coulter, Krefeld, Germany). EDTA values were set to 0 and all data were normalized to total human integrin β2 intensities.

KIM127 antibody was labeled using the Alexa Fluor 647 antibody labeling kit (Thermo Fisher Scientific) following the manufacturer’s instructions.

### Platelet Integrin Activation, Aggregation, Spreading, and Microvascular Thrombosis

Platelet integrin activation, aggregation and spreading were assessed as previously described (17). The following agonists were used for these assays: Chrono-Par-Thrombin, Chrono-Par-ADP, Chrono-Par-Collagen (Probe & go Labordiagnostica GmbH, Lemgo, Germany), U46619 (Enzo Life Sciences GmbH, Lörrach, Germany) and Collagen-related peptide (CRP; kindly provided by Prof. Siess, LMU, Munich). Intravital microscopy of the cremaster to measure thrombus formation after photochemical injury *in vivo* was performed as described earlier (17, 31, 32).

### Neutrophil Trafficking *In Vivo*

Neutrophil rolling, adhesion and extravasation *in vivo* was analyzed by intravital microscopy and subsequent histological analysis of TNF-α-stimulated cremaster muscle venules as previously described (17).

### T Cell Homing FACS Assay and *In Vivo* Imaging

T cell homing was assessed by adoptive transfer experiments and subsequent FACS analysis as previously described (14).

For *in vivo* imaging of T cell trafficking to the lymph node CD4^+^ T cells were enriched from spleens by negative selection. Briefly, spleens were incubated with biotinylated antibodies to Gr-1, B220, CD8, Ter-119 and F4/80 and subsequently with anti-biotin microbeads (Miltenyi Biotec, Bergisch Gladbach, Germany). Antibody bound cells were depleted using MS columns placed in a MidiMACS Separator (Miltenyi Biotec). Mice were injected intravenously with a 1:1 mixture of CFSE and Far Red labelled WT and Tln1^3mut^, Riam^-/-^ or DM cells. Preparation and imaging of the popliteal lymph node vasculature and data analysis were performed as described earlier (33).

### Statistical Analysis

Data are presented as mean ± 95% confidence interval. ANOVA followed by Tukey’s multiple comparison test was performed to determine statistical significance for comparison of data sets using Prism9 (GraphPad Software). Differences between groups were considered statistically significant if p<0.05.

## Results

To assess a potential cooperative function of the direct Rap1/talin and the Rap1/Riam/talin pathways on integrin regulation in hematopoietic cells *in vivo*, we crossed Rap1 binding-deficient talin knock-in (Tln1^3mut^) and Riam-deficient (Riam^-/-^) mouse lines (14, 17) to generate Tln1^3mut^/Riam^-/-^ DM mice. These compound mutant mice are born at normal Mendelian ratio, are viable and exhibit no overt phenotype, similar to the single mutant mouse strains. An analysis of their peripheral blood revealed comparable cell counts between WT and Tln1^3mut^ mice, whereas leukocyte numbers were strongly increased in Riam^-/-^ mice and tended to be even higher in DM mice (**Table S1**).

Even though previous studies revealed that Riam is dispensable for platelet integrin activation and function (14, 15, 19), we wondered whether the mild integrin defect of Tln1^3mut^ platelets is due to some functional rescue by the Riam pathway (17). Before we addressed this experimentally, we first showed comparable expression of talin, Rap1 and kindlin-3 in platelets from WT, Tln1^3mut^, Riam^-/-^ and DM mice by Western blot analyses, and confirmed lack of RIAM expression in Riam^-/-^ and DM platelets (**Figure S1A**). In addition, flow cytometric analyses indicated similar integrin surface levels between wildtype and mutant platelets (**Figure S1B**). We then performed functional assays and analyzed αIIbβ3 integrin activity by conformation specific antibody (JON/A) and fibrinogen binding, agonist-induced platelet aggregation and platelet spreading on fibrinogen upon thrombin stimulation (**Figures S1C–F**). In sum, these experiments confirmed that Riam is not involved in the regulation of platelet integrins, which is also corroborated by the similar defects measured in DM and Tln1^3mut^ platelets. Even *in vivo* microvascular thrombosis assays in cremaster venules and arterioles revealed a similar delay in thrombus formation in response to photochemical injury in Tln1^3mut^ and DM mice, whereas no defect was measured in Riam^-/-^ mice (**Figures S1G, H**). Altogether, our data corroborated the dispensable role of Riam and showed no synergistic action of the Rap1/talin and Rap1/Riam/talin pathways on talin-mediated platelet integrin regulation.

**Figure 1:**
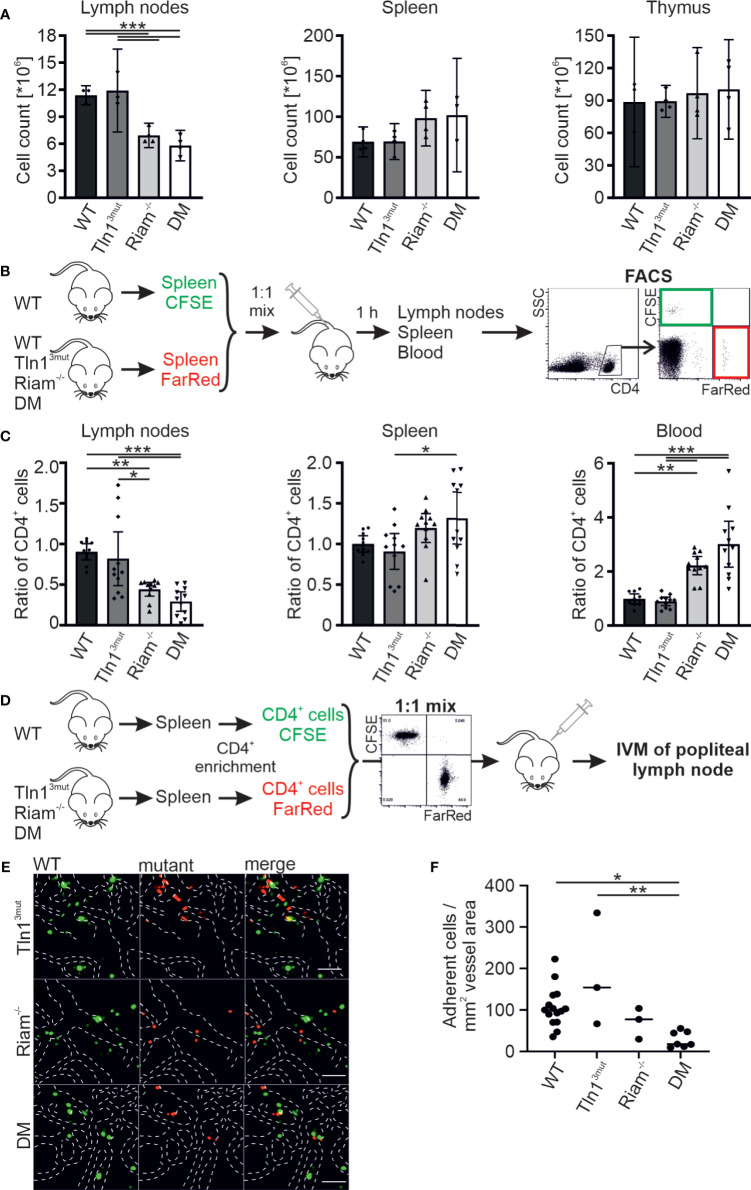
The Rap1/talin and Rap1/Riam/talin pathways regulate T cell homing *in vivo*. **(A)** Cellularity of lymph nodes, spleens and thymi isolated from WT, Tln1^3mut^, Riam^-/-^ and DM mice (N = 4 mice). **(B, C)** T cell lymph node homing assessed in adoptive transfer experiments. A 1:1 mixture of CFSE-stained WT control and FarRed-stained WT, Tln1^3mut^, Riam^-/-^ or DM splenocytes (or swapped dyes) was injected into the tail vein of WT recipients. Ratios of CFSE and FarRed-stained CD4^+^ cells in different organs were measured 1 h after injection by FACS analysis. **(C)** Ratios of transferred WT, Tln1^3mut^, Riam^-/-^ and DM to control cells obtained in lymph nodes, spleen and blood of recipient animals (N = 10/11/11/11). **(D–F)** Adhesion of CD4^+^ T cells assessed *in vivo* by confocal intravital microscopy of the lymph node vasculature after adoptive transfer. **(D)** Schematic overview of the experiment. Recipient animals were injected with a 1:1 mix of CFSE stained WT and FarRed stained Tln1^3mut^, Riam^-/-^ or DM pre-enriched CD4^+^ T cells (or swapped dyes) and subjected to intravital microscopy. **(E)** Exemplary confocal images of WT (green) and Tln1^3mut^, Riam^-/-^ or DM (red) cells in the popliteal lymph node vasculature. Blood vessel borders are highlighted by dashed white lines, scale bars: 50 µm. **(F)** Number of adherent cells normalized by the blood vessel surface area (N=12/3/3/6). All values are given as mean ± 95% confidence interval. Statistical significance was assessed using One-way ANOVA followed by Tukey’s multiple comparison test. *p < 0.05, **p < 0.01, ***p < 0.001.

In contrast to the negligible role of Riam for platelet integrin regulation, the leukocyte specific β2 integrin family critically depends on Riam (14, 15). We have also shown previously that this integrin class is regulated *via* the direct Rap1/talin pathway (17). To investigate, whether the two talin recruitment pathways synergize to fine-tune leukocyte integrin activity, we used T cell homing into the lymph node as model, since this process, as we have previously reported, is β2 integrin and RIAM dependent (14). First, we determined lymph node cellularity of the different mouse lines under steady state conditions and found similar numbers in WT and Tln1^3mut^ mice. In line with the crucial role of Riam for β2 integrin function we measured a reduced lymph node cellularity in Riam^-/-^ mice, which was somewhat enhanced in DM mice. At the same time, we counted slightly increased numbers of splenocytes in Riam and DM mice, whereas thymus cellularity was unchanged in all four mouse lines (**Figure 1A**). To investigate, whether defective T cell homing contributes to the reduction in lymph node cellularity, we performed adoptive transfer experiments. Upon injection of a 1:1 mixture of CFSE-stained WT and FarRed-stained WT, Tln1^3mut^, Riam^-/-^ or DM splenocytes (dyes were switched between experiments) into WT recipient mice, we found a reduction in lymph node homing capacity for Riam^-/-^ and DM CD4^+^ T cells, which was most pronounced for DM cells (**Figures 1B, C**). Consistent with their reduced homing capacity, we detected an increased number of Riam^-/-^ and DM CD4^+^ T cells in the blood and spleen of the recipient animals, with highest numbers found in DM mice (**Figure 1C**). In a parallel experiment, we directly monitored lymphocyte homing into popliteal lymph nodes by intravital microscopy. Therefore, we isolated splenic CD4^+^ T cells from WT and mutant mice, stained them with either CFSE or FarRed and injected a 1:1 mixture into WT recipient mice. The number of adherent cells in relation to the vascular surface area was then determined within the lymph node by intravital imaging (**Figure 1D**). This experiment revealed that less Riam^-/-^ and DM T cells adhered to the lymph node vasculature compared to WT and Tln1^3mut^ T cells (**Figures 1E, F**). In sum, these data suggest a dominant role of the Rap1/Riam/talin pathway in the regulation of T cell adhesion to the vasculature during lymph node homing. Even though no significant difference in T cell homing to the lymph nodes was measured between Riam^-/-^ and DM T cells, the DM T cells consistently showed the strongest defects in both experiments.

**Figure 2:**
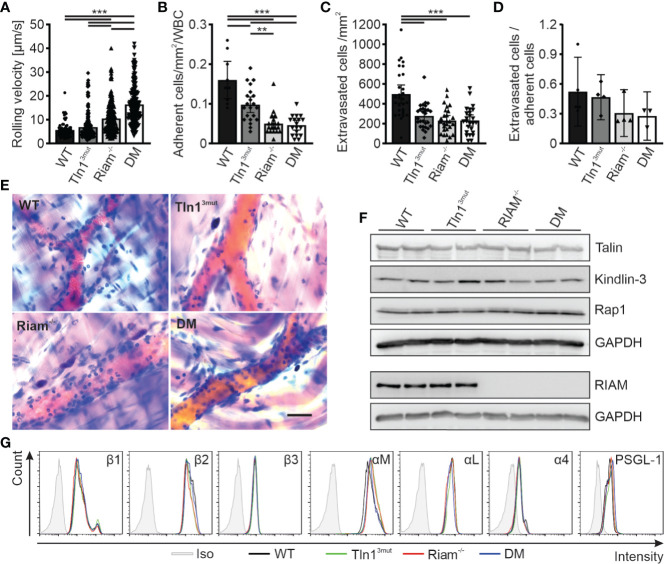
Neutrophil rolling, adhesion and extravasation are impaired when the Rap1/talin and Rap1/Riam/talin pathways are blocked. **(A–E)** *In vivo* leukocyte rolling velocity **(A)**, adhesion efficiency **(B)**, extravasation **(C)** and extravasation efficiency **(D)** analyzed in cremaster muscle venules of WT, Tln1^3mut^, Riam^-/-^ and DM mice 2 h after intrascrotal TNFα injection. Rolling velocity and adhesion efficiency were assessed using intravital microscopy. Extravasation was analyzed by counting the number of perivascular cells in Giemsa stained cremaster muscle whole mounts (N = 3-6 vessels per mouse in 2/4/4/4 mice, 5/4/4/4 mice for **D**). **(E)** Representative images of Giemsa stained cremaster muscle whole mounts, scale bar: 30 µm. **(F)** Analysis of talin, kindlin-3, Rap1 and Riam expression in WT, Tln1^3mut^, Riam^-/-^ and DM neutrophils by western blot. GAPDH was used as loading control. **(G)** Integrin β1, β2, β3, αM, αL and α4 as well as PSGL-1 and L-Selectin surface levels on WT, Tln1^3mut^, Riam^-/-^ and DM neutrophils assessed by FACS analysis. Values represent mean ± 95% confidence interval. Statistical significance was assessed using One-way ANOVA followed by Tukey’s multiple comparison test. **p < 0.01, ***p < 0.001.

We next chose another primarily β2 integrin dependent process to study the significance and relative impact of the Rap1/Talin and Rap1/RIAM/Talin pathways for leukocyte trafficking. Intravital microscopy of the inflamed mouse cremaster muscle represents an excellent method to investigate the different steps of the leukocyte adhesion cascade, such as leukocyte rolling, adhesion and extravasation. In accordance with our previous studies on Tln1^3mut^ and Riam^-/-^ mice (14, 17), Tln1^3mut^ mice showed reduced numbers of adherent and perivascular cells, while their neutrophil rolling velocities were hardly affected (**Figures 2A–E**, **Videos S1****–****4**). Riam^-/-^ neutrophils showed strongly reduced adhesion and extravasation (**Figures 2A–E**, **Videos S1****–****4**). This severe phenotype was not further aggravated in DM mice. As the extravasation efficiency is not significantly reduced between the groups, we conclude that the reduced number of extravasated cells is primarily caused by their defective adhesion (**Figure 2D**). However, the already strongly increased rolling velocity of RIAM^-/-^ neutrophils was additionally markedly increased in DM animals (**Figure 2A**). Of note, talin1, kindlin-3 and Rap1 expression (**Figure 2F**) as well as surface levels of relevant integrins, L-selectin and PSGL-1 (**Figure 2G**) were comparable in neutrophils derived from mice of all four genotypes. These *in vivo* studies suggest that loss of either Rap1/talin or Rap1/Riam/talin pathway impaired leukocyte adhesion and extravasation and that a combined loss of both talin recruitment pathways drastically affects integrin-mediated leukocyte rolling.

**Figure 3:**
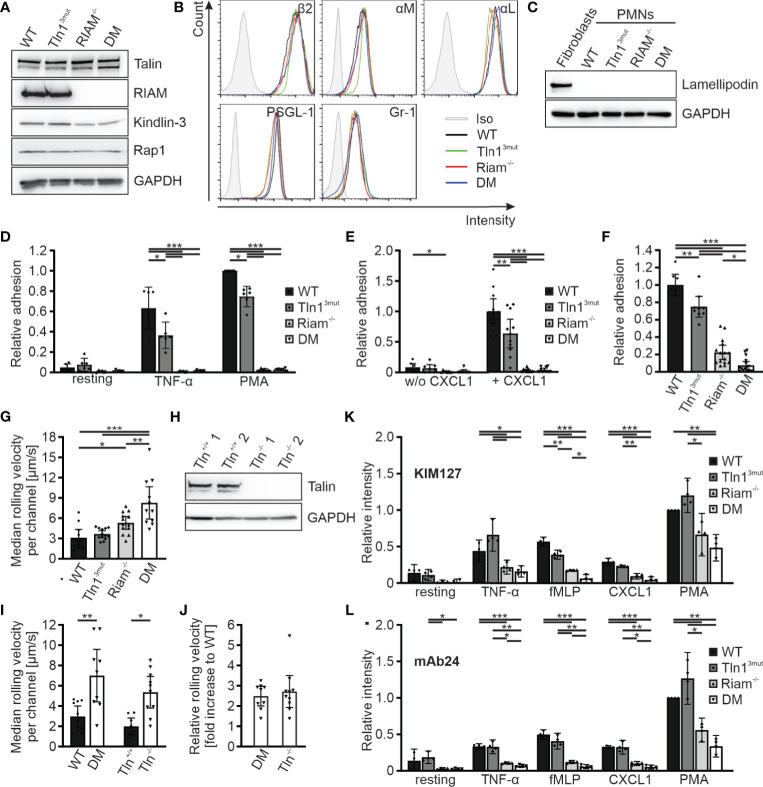
Reduced integrin activity and function in Hoxb8 cell-derived Tln^3mut^, Riam^-/-^ and DM neutrophils. **(A)** Western blots showing Talin, Riam, kindlin-3 and Rap1 expression in Hoxb8 cells derived from WT, Tln1^3mut^, Riam^-/-^ and DM mice. GAPDH served as loading control. **(B)** FACS histograms showing surface levels of the integrin subunits β2, αM and αL as well as PSGL-1 and Gr-1 of WT (black), Tln1^3mut^ (green), Riam^-/-^ (red) and DM (blue) Hoxb8-derived neutrophils. **(C)** Expression of lamellipodin in Hoxb8-derived neutrophils assessed by Western blot. Mouse embryonic fibroblast lysate served as positive control, GAPDH as loading control. **(D)** Relative adhesion of WT, Tln1^3mut^, Riam^-/-^ and DM Hoxb8-derived neutrophils on ICAM-1 either unstimulated (resting) or upon stimulation with TNFα or PMA under static conditions (N = 6). **(E–G)** Rolling and adhesion behavior of WT, Tln1^3mut^, Riam^-/-^ and DM Hoxb8-derived neutrophils assessed in flow chamber experiments. **(E)** Relative adhesion under flow at a wall shear rate of 1 dyne/cm^2^ on P-selectin and ICAM-1 (N = 8 chambers in 4 experiments) or P-selectin, ICAM-1 and CXCL1 coated surfaces (N = 12/11/12/10 chambers in 4 experiments). **(F)** Relative adhesion under flow at a wall shear rate of 0.3 dyne/cm^2^ on P-selectin, ICAM and CXCL1 coated surfaces: WT values are set to 1 (N = 8/9/15/16 chambers in 3 experiments). **(G)** Rolling velocities of Hoxb8-derived neutrophils at a shear rate of 1 dyne/cm^2^ (N = 11/13/13/11 chambers in 4 experiments). **(H)** Western blot analysis of Talin and GAPDH expression in Tln1^+/+^ and Tln1^-/-^ Hoxb8 clones. **(I, J)** Median rolling velocities of WT and DM as well as Tln1^+/+^ and Tln1^-/-^ Hoxb8-derived neutrophils assessed in P-selectin, ICAM-1 and CXCL1 coated flow chambers under 1 dyne/cm^2^ shear stress. Results are shown as absolute values **(I)** and fold rolling velocity increase of DM and Tln^-/-^ cells to respective WT controls **(J)**. Two Tln1^+/+^ and Tln1^-/-^ clones were analyzed (N = 10/9/8/10 chambers in 2 experiments). **(K, L)** FACS analyses of integrin β2 activation by measuring staining intensities of conformation specific antibodies KIM127 **(K)** and mAb24 **(L)** normalized to total integrin β2 levels on Hoxb8-derived neutrophils expressing human β2 integrin either in resting state or in response to TNF-α, fMLP, CXCL1 or PMA. Values of PMA-stimulated WT cells were set to 1 (N = 4 experiments). All values represent mean ± 95% confidence interval. Statistical significance was assessed using ANOVA followed by Tukey’s multiple comparison test. *p < 0.05, **p < 0.01, ***p < 0.001.

In order to decipher the molecular roles of the two talin recruitment pathways in an *in vitro* cell culture system, we generated Hoxb8 cells from the four mouse strains. Hoxb8 cells are immortalized hematopoietic progenitor cells generated from murine bone marrow by expression of a retrovirally-delivered estrogen-regulated form of the transcription factor Hoxb8. Notably, in the presence of Flt3L these cells retain the capacity to differentiate into myeloid and lymphoid cells *in vitro*, which resemble phenotypically and functionally their primary counterparts (27). WT, Tln1^3mut^, Riam^-/-^ and DM Hoxb8 cells were differentiated into neutrophil-like cells by adding SCF and G-CSF to the culture medium and used for the ensuing assays. These neutrophil-like cells were comparable with regard to their talin1, kindlin-3, Riam and Rap1 protein levels as well as their surface levels of integrin subunits β2, αM, αL, and Gr-1 and PSGL-1 (**Figures 3A, B**). Moreover, the expression of the second mammalian MRL protein family member lamellipodin was not upregulated in response to Riam deficiency (**Figure 3C**). First, we performed static adhesion assays on the β2 integrin ligand ICAM-1, which showed significantly reduced adhesion of Tln1^3mut^ neutrophil-like cells in response to TNF-α and PMA, similar to the reported adhesion defect of primary Tln1^3mut^ neutrophils (17), and almost complete loss of adhesion of Riam^-/-^ and DM cells (**Figure 3D**). Next, we studied cell adhesion under flow in P-selectin, ICAM-1 and CXCL1 coated flow chambers. Consistent with our previous experiments with primary Tln1^3mut^ and Riam^-/-^ neutrophils (14, 17), we measured a mild adhesion defect of Tln1^3mut^ Hoxb8-derived neutrophils compared to an almost complete loss of adhesion of Riam^-/-^ and DM neutrophils at a wall shear stress of 1 dyne/cm^2^. Of note, adherent cells were hardly observed for all genotypes on surfaces coated with P-selectin and ICAM-1 only, which indicates that leukocyte adhesion is triggered by chemokine induced integrin inside-out activation (**Figure 3E**). To test, whether neutrophil adhesion can occur in the absence of the Rap1/Riam/talin pathway under low flow conditions, we compared WT, Tln1^3mut^, Riam^-/-^ and DM Hoxb8-derived neutrophils in flow chamber experiments at a shear rate of 0.3 dyne/cm^2^. This experiment revealed indeed that in the absence of Riam some neutrophils adhered to the flow chambers, which became significantly less when the Rap1/talin pathway was additionally impaired (**Figure 3F**). In other words, the Rap1/talin pathway is sufficient to induce cell adhesion at least under low flow. This result further strengthened our *in vivo* findings that both pathways significantly contribute to talin-induced integrin-mediated leukocyte adhesion.

We then analyzed rolling of Hoxb8-derived neutrophils in P-selectin, ICAM1, CXCL1 coated flow chambers. While most leukocyte rolling is mediated *via* selectins, interactions of integrins with their ligands support leukocyte rolling and further decelerate their rolling velocity (integrin-mediated slow rolling) (2, 4, 34). Consistent with the *in vivo* studies in the cremaster muscle (**Figure 2A**), rolling velocities were significantly increased in Riam^-/-^ cells and considerably higher in DM neutrophils, which clearly indicate a synergistic effect of both talin recruitment pathways on integrin-mediated leukocyte rolling (**Figure 3G**). Since we have previously shown that talin-deficient neutrophils roll faster than Riam knockout neutrophils (14), we wondered whether a concurrent loss of both talin recruitment pathways will recapitulate a talin-knockout phenotype. Thus, we generated talin1 deficient Hoxb8 clones (Tln1^-/-^) using the CRISPR/Cas9 technique and confirmed talin1 deficiency by Western blot analysis (**Figure 3H**). We then differentiated WT and DM Hoxb8 cells as well as two Tln1^-/-^ and two equally treated, but non-targeted WT clones (Tln^+/+^) into neutrophils, and assessed their rolling velocities in flow chamber experiments. Strikingly, the median rolling velocities of DM and Tln1^-/-^ neutrophils are similarly (~2.5-fold) increased compared to their respective control cells (**Figures 3I, J**). These experiments strongly suggest that the Rap1/talin and the Rap1/Riam/talin pathways are the key talin recruitment pathways involved in the induction of β2 integrin-mediated leukocyte rolling.

Integrin-mediated slow rolling and firm adhesion are conveyed by the intermediate-affinity extended-closed conformation and the high-affinity, extended-open conformation, respectively. We finally wanted to clarify, whether stimulation of the two talin recruitment pathways translates into conformational changes of the integrin ectodomain. However, as conformation specific antibodies exist only for human but not mouse β2 integrins, we induced a β2 integrin knock-out in WT, Tln1^3mut^, Riam^-/-^ and DM Hoxb8 cells using the CRISPR/Cas9 technology and reconstituted the cells with the human β2 integrin ortholog by retroviral transduction. These humanized Hoxb8 cells were then differentiated into neutrophils, stimulated with TNF-α, fMLP, CXCL1 and PMA and stained with the extended conformation specific antibody clone KIM127 (**Figure 3K**) or high-affinity conformation specific antibody clone mAb24 (**Figure 3L**). These experiments revealed a strong reduction in KIM127 and mAb24 binding to activated Riam^-/-^ cells, which was further reduced in DM cells (**Figures 3K, L**). In sum, these data indicate that the two pathways converge in triggering conformational changes of the integrin towards an active conformation.

## Discussion

The recruitment of cytoplasmic talin to the plasma membrane represents an essential and complex process during integrin activation, which apparently proceeds in different ways dependent on cell type and integrin involved. For example, we recently showed that in fibroblasts the direct Rap1/talin pathway plays a central role in the regulation of integrin activation, whereas the talin/Riam interaction is irrelevant in these cells (23). Similarly, in platelets, the Rap1/talin pathway is dominant and regulates integrin activation through cooperative binding of Rap1 to the F0 and F1 domains of talin (24, 25). In this study, we corroborated a recent study showing that Riam is not involved in platelet integrin regulation. However, this work used a Rap1 binding mutant that still allowed weak binding of Rap1 to the talin F0 domain (17, 19).

In our current study, we mainly focused on leukocyte β2 integrins. Here we wanted to address the question whether talin recruitment to the plasma membrane during β2 integrin activation (inside-out signaling) can occur in the absence of Riam and whether the direct Rap1/talin pathway also contributes. This question arises, because β2 integrin function of Riam knockout mice is almost completely abolished similar as in the absence of talin. On the other hand, Rap1/talin binding mutants show a weak β2 integrin defect. Indeed, our studies show that Riam plays a dominant role in controlling β2 integrin function and loss of Riam results in a strong defect in leukocyte adhesion and extravasation and results in strongly impaired lymphocyte homing *in vivo*. Interestingly, this defect is further exacerbated by a loss of Rap1/talin interaction, which is reflected by a markedly increased leukocyte rolling velocity *in vivo* and further reduced leukocyte adhesion in *in vitro* flow chamber experiments of DM cells. This clearly indicates a synergy of both pathways in the regulation of β2 integrins. Why Riam primarily regulates the activity of β2 integrins and not, for example, integrins of fibroblasts or platelets, may be due only in part to the low expression of Riam in these cells, but more likely to the formation of a Riam-dependent β2 integrin-specific activation complex that is only formed in certain hematopoietic cells. This hypothesis is also supported by the fact that in the absence of Riam, β2 integrins on regulatory T cells can be activated by the Riam paralogue lamellipodin, which is highly expressed in these cells (35). In contrast, the Rap1/talin pathway appears to be a general mechanism for the regulation of all integrin classes, as reflected by studies in various model organisms and cell types such as *Dictyostelium*, fly, mouse, platelets, fibroblasts and leukocytes (17, 19, 21, 22). Apparently, two Rap1-mediated pathways with different direct effectors, Riam and Talin, have been established to control integrin activity: The Rap1/talin pathway that applies equally to all integrin classes independent of the cell type and the Rap1/Riam/talin pathway that is specific to β2 integrins. While the two pathways differ mechanistically in that the Riam N-terminus binds to the talin F3 and multiple sites of the talin rod and Rap1 binds directly to the talin F0 and F1 domains, ultimately both pathways lead to the recruitment of talin to the plasma membrane.

While the effect of the Rap1/talin pathway is relatively weak on β2 integrin-mediated adhesion, we found a strong impact on integrin-mediated rolling in DM cells, which requires integrin ectodomain extension adopting an intermediate ligand affinity conformation that transiently interacts with ligands on the vascular surface. That this process is possible when either pathway is blocked but results in a rolling defect similar to that seen in talin-null cells when both pathways are blocked, suggests that both pathways are used for the initial recruitment of talin to the membrane and induction of the extended conformation. Firm anchoring of the cells to the vascular wall or flow chamber surface, on the other hand, mainly relies on the Riam-dependent pathway, whereas the Rap1/talin pathway only allows adhesion at low shear forces. So, what distinguishes the two pathways and why does β2 integrin function strongly depend on the Rap1/Riam/talin pathway while other integrins do not? An explanation could be that the Rap1/talin pathway only contributes to talin activation by recruiting it to the plasma membrane, while the Rap1/Riam/talin pathway plays a dual role. Riam recruits talin to the membrane by interacting with the R3 domain and displaces autoinhibitory talin head-rod interactions by interacting with the F3 domain (36). Although talin recruitment to the plasma membrane through both pathways indirectly facilitates conformational activation of talin through attractive and repulsive interactions of the talin head and rod domains with negatively charged membrane lipids *via* a pull-push mechanism (37), the release of talin autoinhibition by Riam may be particularly important for efficient β2 integrin activation. Moreover, firm cell adhesion requires linkage of integrins to the actin cytoskeleton, which could either indirectly occur *via* subsequent exchange of Riam with vinculin upon talin recruitment or directly *via* the C-terminal ENA/Vasp binding sites of Riam. The ability of this complex to promote the formation of cell protrusions and targeting of integrins to their tips might be a further explanation for the dominant role of Riam during leukocyte adhesion (11, 38–40).

It is important to note that the rather weak defects in rolling, adhesion and extravasation observed in Tln^3mut^ mice and Hoxb8-cells arise from an incomplete block of Rap1-binding, as only the Rap1-binding site within talin F0 domain was mutated here. Interaction with the Rap1-binding site in the subsequent talin F1 domain, which acts synergistically with the binding site within the F0 domain, is therefore still possible. The importance of both binding sites together is highlighted by the relatively mild phenotypes of mice in which only one of the two Rap1 binding sites was mutated, while double mutants are early embryonic lethal (17, 19, 24, 25). Therefore, we potentially underestimate the influence of the Rap1/talin pathway in our study due to compensation by the second Rap1 binding site.

Overall, our study shows for the first time that a concerted action of the Rap1/talin and the Rap1/Riam/talin pathways efficiently recruits talin from the cytoplasm to β2 integrin adhesions during the initial step of β2 integrin inside-out signaling.

## Data Availability Statement

The raw data supporting the conclusions of this article will be made available by the authors, without undue reservation.

## Ethics Statement

The animal study was reviewed and approved by District government of Upper Bavaria, Munich, Germany.

## Author Contributions

TB and MM conceived the study, designed experiments, interpreted data, and wrote the manuscript with contributions from all other authors. TB, SK, IR, JW, RP, LM, and MM performed and analyzed experiments. SM-W, CR, CS, and MS provided vital reagents and critical expertise. All authors contributed to the article and approved the submitted version.

## Funding

This work was supported by the Deutsche Forschungsgemeinschaft (SFB914 TP A01, B01, B03, B09 and Z03), eRARE/BMBF to MM (LADOMICs consortium) and the Max Planck Society.

## Conflict of Interest

The authors declare that the research was conducted in the absence of any commercial or financial relationships that could be construed as a potential conflict of interest.

## Publisher’s Note

All claims expressed in this article are solely those of the authors and do not necessarily represent those of their affiliated organizations, or those of the publisher, the editors and the reviewers. Any product that may be evaluated in this article, or claim that may be made by its manufacturer, is not guaranteed or endorsed by the publisher.
