# Supplementary material for: Binding of Rap1 and Riam to Talin1 Fine-Tune β2 Integrin Activity During Leukocyte Trafficking
Source: Front Immunol. 2021 Aug 19;12:702345. doi: 10.3389/fimmu.2021.702345 (PMC8417109; doi:10.3389/fimmu.2021.702345)
Supplement: Supplementary file 1 [file DataSheet_1.pdf]

Supplementary material

Table S1: Peripheral blood cell counts of WT, Tln1^{3mut}, Riam$^{-/-}$ and double mutant (DM) mice (N=15/15/9/11). Values are given as mean ± 95% confidence interval.

	**WT** (N=15)	**Talin^{3mut}** (N=15)	**Riam$^{-/-}$** (N=9)	**DM** (N=11)
**Platelets [K/µl]**	756 ± 129	726 ± 132	758 ± 210	627 ± 168
**Mean platelet volume [fL]**	6.68 ± 0.76	6.57 ± 0.59	7.31 ± 0.76	7.81 ± 0.87
**White blood cells [K/µl]**	7.22 ± 1.31	7.98 ± 2.15	13.51 ± 2.16	14.46 ± 2.86
**Neutrophils [K/µl]**	1.42 ± 0.46	1.06 ± 0.39	3.08 ± 1.10	3.22 ± 1.49
**Lymphocytes [K/µl]**	5.57 ± 1.27	6.71 ± 1.78	10.02 ± 1.66	10.75 ± 2.37
**Monocytes [K/µl]**	0.10 ± 0.05	0.08 ± 0.04	0.05 ± 0.02	0.15 ± 0.08
**Eosinophils [K/µl]**	0.12 ± 0.05	0.13 ± 0.07	0.35 ± 0.07	0.33 ± 0.14
**Basophils [K/µl]**	0.01 ± 0.01	0.01 ± 0.00	0.00 ± 0.00	0.01 ± 0.01
**Red blood cells [M/µl]**	9.95 ± 0.28	10.01 ± 0.30	10.60 ± 0.25	10.10 ± 0.30
**Hemoglobin [g/dL]**	14.97 ± 0.35	15.15 ± 0.46	14.98 ± 0.29	14.88 ± 0.56
**Hematocrit [%]**	47.91 ± 1.46	48.91 ± 1.53	48.88 ± 1.11	48.05 ± 1.89

Figure S1

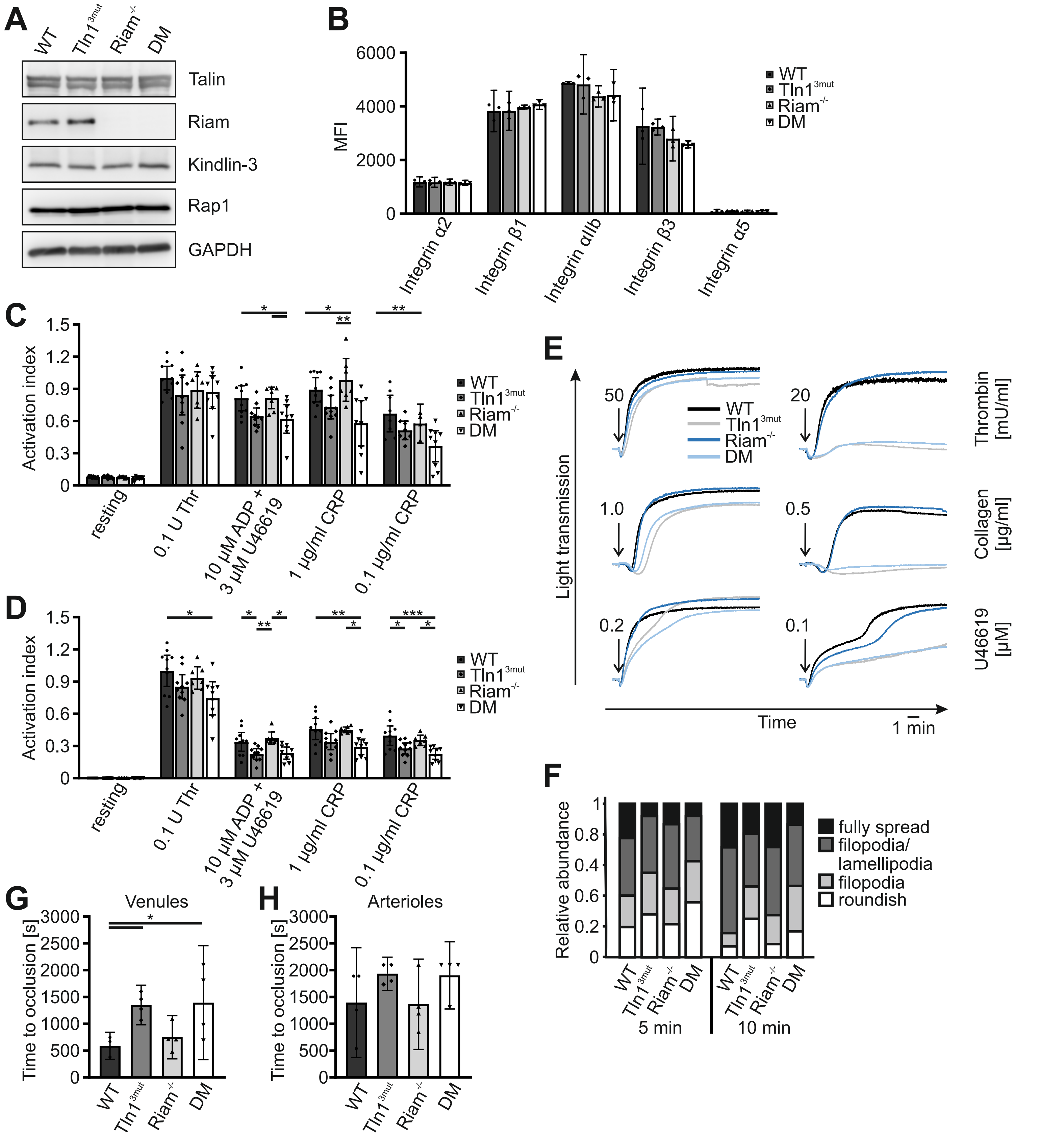

Supplementary figure 1: Platelet integrins are regulated via the Rap1/talin pathway but not the Rap1/Riam/talin pathway. (A) Western blots showing expression levels of Talin, Riam, kindlin-3 and Rap1 in WT, Tln1^{3mut}, Riam$^{-/-}$ and DM platelets. GAPDH served as loading control. (B) Surface expression of integrin subunits α2, β1, αIIb, β3 and α5 on WT, Tln1^{3mut}, Riam$^{-/-}$ and DM platelets measured by FACS analysis (N=3 mice). (C/D) Integrin αIIbβ3 activation on WT, Tln1^{3mut}, Riam$^{-/-}$ and DM platelets in response to different agonists assessed by (C) active integrin αIIbβ3 specific JON/A antibody binding assay and (D) AlexaFluor647 labeled fibrinogen binding assay. Staining intensities were measured by FACS and normalized to total integrin αIIb levels (N=10/10/7/9 mice in 3 experiments). (E) Platelet aggregation of WT, Tln1^{3mut}, Riam$^{-/-}$ and DM platelets in response to thrombin, collagen and U46619 measured by densitometry. (F) Platelet spreading on fibrinogen coated surfaces in response to 0.01 U/ml thrombin after 5 and 10 min. Values represent the frequencies of platelets adopting a roundish shape, forming filopodia, forming filopodia and lamellipodia or being fully spread for each genotype. (G/H) Thrombus formation monitored by intravital microscopy of cremaster muscle venules (G) and arterioles (H) upon photochemical injury. Values represent the time from injury to complete blood vessel occlusion (N=4 mice). All values represent mean ± 95% confidence interval. Statistical significance was assessed using One-way ANOVA followed by Tukey's or Dunnett's (G/H) multiple comparison test. * $p<0.05$, ** $p<0.01$, *** $p<0.001$.

Supplementary videos 1-4: Representative videos from intravital microscopy of inflamed cremaster muscles of WT, $Tln1^{3mut}$, $Riam^{-/-}$ and DM mice 2 hours after TNF-α injection, scale bars: 30 µm.
